# Physiologically‐based pharmacokinetic modelling of long‐acting injectable cabotegravir and rilpivirine in pregnancy

**DOI:** 10.1111/bcp.16006

**Published:** 2024-02-10

**Authors:** Shakir Atoyebi, Fazila Bunglawala, Nicolas Cottura, Sandra Grañana‐Castillo, Maiara Camotti Montanha, Adeniyi Olagunju, Marco Siccardi, Catriona Waitt

**Affiliations:** ^1^ University of Liverpool Liverpool UK

**Keywords:** antiretrovirals, PBPK, pregnancy

## Abstract

**Aims:**

Long‐acting cabotegravir and rilpivirine have been approved to manage HIV in adults, but data regarding safe use in pregnancy are limited. Physiologically‐based pharmacokinetic (PBPK) modelling was used to simulate the approved dosing regimens in pregnancy and explore if *C*_trough_ was maintained above cabotegravir and rilpivirine target concentrations (664 and 50 ng/mL, respectively).

**Methods:**

An adult PBPK model was validated using clinical data of cabotegravir and rilpivirine in nonpregnant adults. This was modified by incorporating pregnancy‐induced metabolic and physiological changes. The pregnancy PBPK model was validated with data on oral rilpivirine and raltegravir (UGT1A1 probe substrate) in pregnancy. Twelve weeks' disposition of monthly and bimonthly dosing of long‐acting cabotegravir and rilpivirine was simulated at different trimesters and foetal exposure was also estimated.

**Results:**

Predicted *C*_trough_ at week 12 for monthly long‐acting cabotegravir was above 664 ng/mL throughout pregnancy, but below the target in 0.5% of the pregnant population in the third trimester with bimonthly long‐acting cabotegravir. Predicted *C*_trough_ at week 12 for monthly and bimonthly long‐acting rilpivirine was below 50 ng/mL in at least 40% and over 90% of the pregnant population, respectively, throughout pregnancy. Predicted medians (range) of cord‐to‐maternal blood ratios were 1.71 (range, 1.55‐1.79) for cabotegravir and 0.88 (0.78‐0.93) for rilpivirine between weeks 38 and 40.

**Conclusions:**

Model predictions suggest that monthly long‐acting cabotegravir could maintain antiviral efficacy throughout pregnancy, but that bimonthly administration may require careful clinical evaluation. Both monthly and bimonthly long‐acting rilpivirine may not adequately maintain antiviral efficacy in pregnancy.

**What is already known about this subject**

- Approved monthly and bimonthly dosing of long‐acting injectable cabotegravir and rilpivirine can achieve target plasma concentrations in nonpregnant adults.
- Pregnancy is known to alter the disposition of many drugs.
- Physiologically‐based pharmacokinetic models can enable predictions of drug exposure in humans.

**What this study adds**

- Monthly long‐acting injectable cabotegravir is predicted to maintain efficacious drug concentration levels regardless of when initiated in pregnancy.
- Pregnancy is predicted to reduce exposure of long‐acting cabotegravir and rilpivirine, particularly when administered bimonthly.
- Bimonthly dosing of long‐acting cabotegravir requires caution in some pregnant women, but both monthly and bimonthly dosing of long‐acting rilpivirine might be better avoided in pregnancy; this PBPK model provides a foundation for future clinical studies.

## 1 INTRODUCTION

As of 2021, the Joint United Nations Programme on HIV/AIDS (UNAIDS) reported that 38.4 million people were living with HIV across the world and about half of these were women and girls.^1^ Many countries around the world have adopted the test‐and‐treat strategy for managing HIV treatment as recommended by WHO.^2^, ^3^ This implementation has contributed to improvement of viral suppression and has been associated with a reduced risk of HIV transmission, better quality of life and increased life expectancy in people living with HIV.^4^, ^5^, ^6^, ^7^ Despite this overwhelming evidence, UNAIDS estimates that only 81% of pregnant women accessed antiretroviral drugs in 2021 to prevent transmission of HIV to their children.^1^

Pregnant women often experience nausea and vomiting and/or difficulty swallowing, which could contribute to the challenges associated with using oral antiretroviral drugs in this population.^8^, ^9^ Frequent oral administration of drugs can also pose different pharmacological and psychosocial challenges when managing a chronic condition like HIV.^10^, ^11^ Reduction in drug adherence is often observed over time with increased risk of therapeutic failure and development of drug resistance.^10^, ^12^ Similarly, some antiretrovirals, such as rilpivirine (RPV), are recommended to be taken after a meal to ensure adequate bioavailability, which might be difficult for some pregnant women.^13^ In contrast, long‐acting formulations have advantages, including significantly reducing pill burden, consequently improving drug adherence and by‐passing various barriers associated with oral administration.^11^, ^14^ These characteristics make them a potential suitable treatment option for pregnant women experiencing difficulties regarding oral administration. Recently, long‐acting injectable (LAI) cabotegravir (CAB) and LAI RPV were approved by the US Food and Drug Administration (FDA) and European Medicines Agency (EMA).^11^ Both LAI CAB and LAI RPV are co‐packaged in separate vials with approved doses of 600 and 400 mg CAB, and 900 and 600 mg RPV, prepared for intra‐muscular injections. The muscular injections are administered after an oral lead‐in phase where oral CAB and oral RPV are administered for almost a month to assess tolerability.

Pregnancy is associated with anatomical, physiological and metabolic changes that influence pharmacokinetics (PKs).^15^ Though intramuscular (IM) administration of antiretrovirals might bypass some effects of pregnancy on oral drug absorption, IM administration of antiretrovirals remain vulnerable to pregnancy effects on drug distribution, metabolism and elimination.^15^, ^16^ For instance, CAB and RPV are mainly metabolized by uridine diphosphate‐glucuronosyltransferase (UGT) 1A1 and cytochrome P450 (CYP) 3A4, respectively, with minor contributions from UGT1A9 for CAB,^14^, ^17^ and studies have suggested that the activities of both enzymes are upregulated in pregnancy.^18^, ^19^ Limited clinical PK data on LAI CAB and LAI RPV during pregnancy at the time of approval by regulatory agencies implies there is inadequate information to guide the dosing of the IM formulations of the drugs in pregnant women.^17^ With LAI CAB and LAI RPV, pregnant women might benefit from the less frequent drug administration and as an alternative regimen unaffected by nausea and vomiting. However, the approved dosing regimen of these drugs in adults could be at risk of reduced drug concentrations in pregnancy, which might fall below the effective plasma concentration thresholds associated with adequate viral suppression. The commonly adopted *C*_trough_ target for CAB is four times the protein‐adjusted‐IC_90_ (4PAIC_90_ = 0.664 μg/mL). For RPV, different *C*_trough_ targets exist, which include the protein‐binding‐adjusted 90% effective concentration (EC_90_) for RPV (PAEC_90_ = 12 ng/mL) and 50 ng/mL (an approximation of its 4PAEC_90_ = 48 ng/mL).^20^, ^21^ Inadequate viral suppression increases the risk of perinatal HIV transmission and the potential development of viral drug resistance. Currently, there are insufficient clinical data on the PKs of LAI CAB and LAI RPV in pregnancy. Moreover, the data suggests that plasma concentrations of oral RPV are reduced in pregnancy, whereas pregnancy was considered as not affecting plasma concentrations of LAI CAB and LAI RPV during the washout period after discontinuation in pregnant women.^21^, ^22^, ^23^, ^24^ The common exclusion of pregnant women from many clinical trials leads to limited clinical data available to guide drug dosing in the pregnant population.^25^ However, computational tools are increasingly employed to predict the impact of pregnancy on PK.^19^, ^26^, ^27^

Physiologically‐based pharmacokinetic (PBPK) models are mechanistic tools capable of representing the mechanisms involved in drug disposition within biological systems. PBPK models employ mathematical equations to integrate biological system information for a population along with drug‐specific parameters towards characterizing the disposition of the drug within the system.^28^ Usually, the anatomical, physiological and demographical data of a population are used to define the biological system parameters. Similarly, the drug‐specific parameters comprise the physicochemical properties of the drug (eg, acid dissociation constant and lipophilicity) and in vitro data on the drug (eg, fraction of unbound drug in plasma and intrinsic enzymatic clearance of the drug).^28^, ^29^ In this study, we developed and validated a pregnancy PBPK model to evaluate if the dosing regimens of LAI CAB and LAI RPV approved for use in adults could maintain the respective *C*_trough_ targets for CAB and RPV during pregnancy. These models were also used to estimate the extent of foetal exposure to the LAI CAB and LAI RPV during pregnancy.

## 2 METHODS

### 2.1 PBPK model description

A full‐body adult PBPK model was developed in SimBiology®, a product of MATLAB® software, version R2019a (MathWorks, Natick, USA, 2019). Initially, a virtual cohort with 100 healthy individuals was simulated for the validation of the nonpregnant adult PBPK model. The ratio of male:female in this virtual cohort was 50:50 to represent the mixed gender in the clinical studies within the nonpregnant population. For the validation of the pregnancy PBPK model (developed from the adult PBPK model; see Section 2.7), another virtual cohort with 100 healthy individuals (100% female) was simulated with the pregnancy PBPK model to represent a pregnant population. For each model prediction, four different virtual cohorts were simulated, each with 100 healthy individuals.

The distribution of other demographic characteristics (eg, age, weight and body mass index) of the virtual cohort was modelled to replicate the individuals in the clinical studies used for the model validation. Organ weights were determined using anthropometric equations previously reported by Bosgra et al.^30^ Organ volumes were calculated from the organ weights and the respective organ densities reported in the literature.^31^ Regional blood flow to organs and tissue were calculated as fractions of the cardiac output.^32^

### 2.2 Oral absorption and LAI administration

Oral drug absorption was modelled using the compartmental absorption and transit model that has been previously described in the literature.^33^ The rate constant of oral drug absorption (*K*a) was determined using the effective drug permeability. The effective drug permeability was either calculated from the polar surface area (PSA) and number of hydrogen bond donors (HBD) of the drug or with the apparent permeability of the drug across caco‐2 cells.^34^

Intestinal clearance of RPV (Cl_gut_) by CYP3A4 was determined using the hepatic intrinsic clearance^35^ and abundance of CYP3A4^36^ in the intestine as previously described in the literature.^34^ Thus, the fraction of the drug escaping intestinal metabolism into the liver was modelled using Equation (1):

(1) $$Fg=\frac{Q\mathrm{gut}}{Q\mathrm{gut}+\left(fu,\mathrm{gut}\times \text{Clgut}\right)}$$

where *Q*_gut_ is the rate of blood flow to the gut (L/h) and *f*_u,gut_ is the unbound fraction of the drug in the gut. *f*_u,gut_ was considered to be 1 in the model.^37^

The release of the LAI drug formulation from the IM depot compartment was modelled as a first‐order reaction,^38^ shown in Equation (2), and the rates of drug release were fitted to available clinical data.^39^, ^40^

(2) Amuscledt=−KIM×AIMdepot,muscle

where *K*_IM,_ is the release rate of drug (h^−1^) from the IM depot, *A*_IM depot, muscle_ is the amount of the drug (mg) in the IM depot within the muscle and *A*_muscle_/d*t* is the amount of drug released/per unit time from the IM depot into the systemic circulation (mg/h).

### 2.3 Model distribution

**FIGURE 1:**
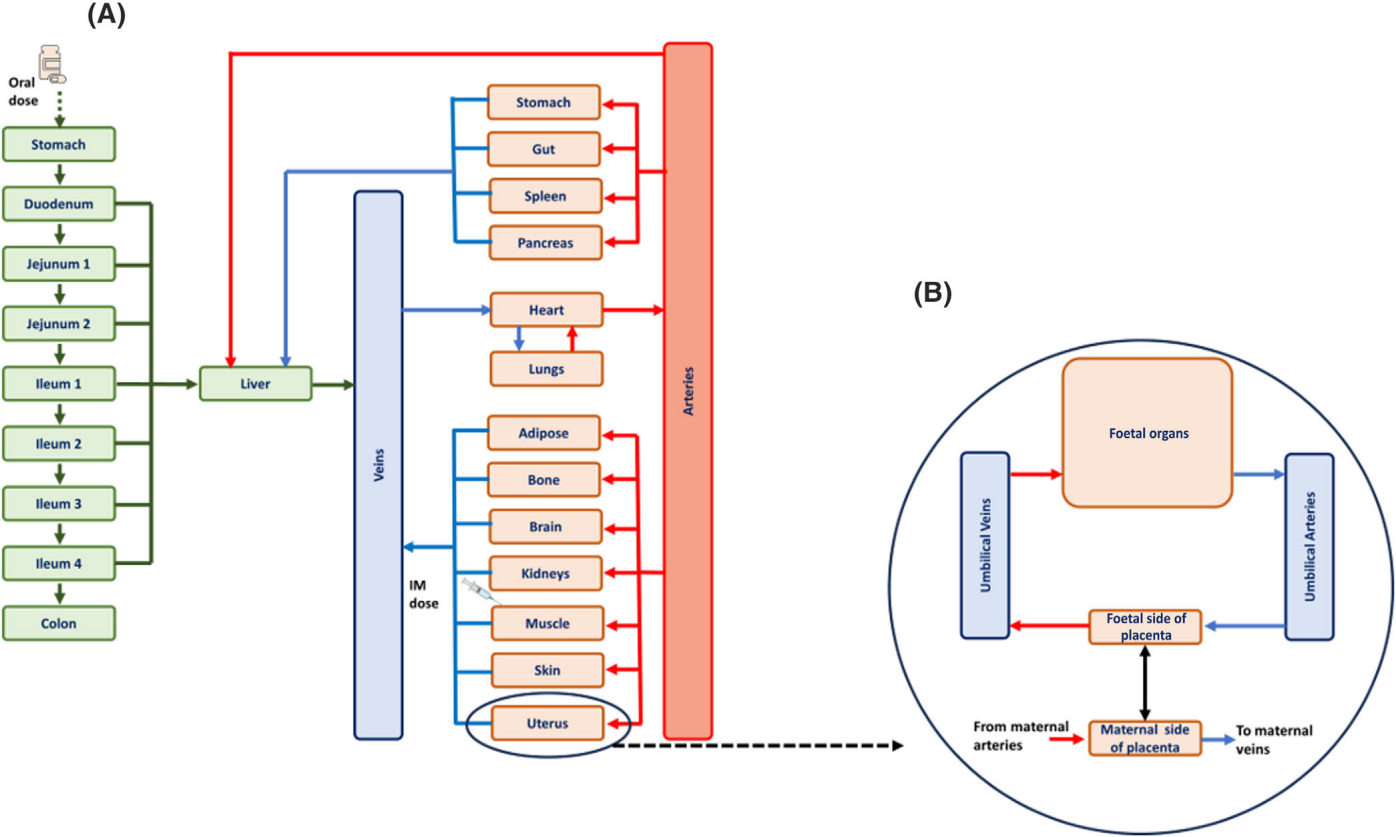
(A) Schematic pregnancy physiologically‐based pharmacokinetic (PBPK) model diagram illustrating organs and tissues as compartments, and blood flows as (blue/red) arrows. IM, intramuscular. (B) Illustration of the foetal compartment as modelled within the uterus.

The pregnancy PBPK model is shown in Figure 1. A foetal component within the female reproductive organ was also included within this pregnancy PBPK model. Major assumptions in the PBPK model include perfusion‐limited drug distribution, well‐stirred distribution and no drug reabsorption from the colon. The volume of drug distribution (*V*_d_) was calculated from the volume and tissue‐to‐plasma ratio of each compartment, as previously described.^41^ The pregnancy effect on the fraction of the unbound drug was also modelled using equations previously described in the literature.^26^, ^42^ Lastly, the activity of drug transporters was not included in the model due to inadequate data for such characterization.

### 2.4 Liver metabolism

The clearance of the drug by the liver was calculated from the intrinsic clearance of the drug by the CYP3A4 enzyme for RPV and by the UGT1A1 and UGT1A9 enzymes for CAB.^43^ The intrinsic clearance of the drug by each enzyme was scaled to the whole of the liver using the microsomal protein content per gram of liver (MPPGL) and the weight of the liver. The intrinsic clearances for RPV and CAB were calculated using Equations (3) and (4), respectively:

(3) $${\mathrm{Cl}}_{\mathrm{CYP}3A4,\text{liver}}={\mathrm{Cl}}_{\mathrm{int},\mathrm{CYP}3A4,\text{liver}}\times \text{abundance}\times \text{MPPGL}\times {\text{weight}}_{\text{liver}}$$

(4) $${\mathrm{Cl}}_{\mathrm{UGT},\text{liver}}={\mathrm{Cl}}_{\mathrm{int},\mathrm{UGT},\text{liver}}\times \text{MPPGL}\times {\text{weight}}_{\text{liver}}$$

where Cl_CYP3A4,liver_ is the hepatic clearance by the CYP3A4 enzyme (L/h), Cl_int,CYP3A4,liver_ (μL/min/pmol) is the intrinsic clearance by a CYP enzyme, Cl_UGT,liver_ is the hepatic clearance by a UGT enzyme (L/h), Cl_int,UGT,liver_ (μL/min/mg) is the intrinsic clearance per milligram of microsomal protein, abundance is the enzyme abundance of CYP3A4 per milligram of microsomal protein (pmol/mg), MPPGL is the weight (mg) of microsomal protein per gram of liver (mg/g) and weight_liver_ is the weight of the liver.^38^

The total hepatic clearance of the drug in the liver (Cl_h_) was used to calculate the fraction of the drug reaching the systemic circulation from the liver, as described in Equation (5):

(5) $$F_{h}=\frac{Q_{h}}{Q_{h}+\left({\mathrm{Cl}}_{h}\times \frac{f\mathrm{up}}{R}\right)}$$

where *F*_h_ is the fraction of the drug escaping hepatic metabolism and entering the systemic circulation from the liver, *Q*_h_ is the rate of blood flow to the liver, Cl_h_ is the total hepatic clearance of the drug in the liver, *f*_up_ is the fraction of the unbound drug in plasma and *R* is the tissue‐to‐plasma partition of the drug in the liver.

### 2.5 Drug parameters

Raltegravir (RAL) was included in this study as a probe substrate for the activity of UGT1A1 in pregnancy. RAL is solely metabolized by UGT1A1 and can be used to validate the ontogeny pf UGT1A1 in both pregnant and nonpregnant populations. Similarly, clinical PK data of RAL in both populations are available. Unlike CAB, clinical PK data for oral RPV in pregnancy are available,^21^, ^22^, ^24^ but there are no available PK data for LAI RPV in pregnancy. Physicochemical properties and in vitro data for the drugs modelled in this study (RPV, RAL and CAB) are listed in Table 1. Renal drug clearance was not implemented in the model as all three drugs are mainly cleared by the liver.

**TABLE 1:** Input drug parameters for the CAB, RAL and RPV models.

Drug parameter	CAB	RAL	RPV
Molecular weight	405	444	366
p*K*a	10.04^43^	6.67^38^	3.26^43^
*R*	0.441^43^	0.6^38^	0.67^43^
Log *P*_o:w_	1.04^43^	0.58^38^	4.32^43^
*f*_up_	0.007^43^	0.17^38^	0.003^43^
HBD	2^43^		
PSA	99.2^43^		
Caco‐2 *P*_app_ × 10^−6^ (cm/s)	12.3^44^	6.6^38^	12^43^
CYP3A4 CL_int_ (μL/min/pmol)			2.04^43^
UGT1A1 CL_int_ (μL/min/mg)	4.5^43^	27.7^45^	
UGT1A9 CL_int_ (μL/min/mg)	2.2^43^	5.5^45^	
IM drug release rate^b^ (h^−1^)	3.406 × 10^−4^		4.5 × 10^−4^
Drug diffusion rate constant across placenta	0.028^b^	0.015^a^	0.027^b^

Abbreviations: CAB, cabotegravir; Cl_int_, intrinsic clearance; CYP, cytochrome P450; *f*_up_, fraction of drug unbound in plasma; HBD, number of hydrogen bond donors; Log *P*_o:w_, partition coefficient between octanol and water; *P*_app_, apparent permeability coefficient; p*K*a, acid dissociation constant; PSA, polar surface area; *R*, blood‐to‐plasma drug ratio; RAL, raltegravir; RPV, rilpivirine; UGT, uridine diphosphate‐glucuronosyltransferase.

^a^ Value fitted in the model using available clinical PK data.

^b^ Value derived using the diffusion constant and Papp of RAL alongside the *P*_app_ of the drug.

### 2.6 Adult PBPK model validation against available clinical data

The oral regimens tested in adults included 30 mg CAB single dosing and 30 mg CAB repeated dosing, 25 mg RPV and 150 mg RPV repeated dosing, and 400 mg RAL repeated dosing. The clinical PK data of single doses of 30 mg of oral CAB were obtained from the study reported by Ford et al, where one single dose of 30 mg of CAB was orally administered to study participants (n = 15).^46^ For the repeated dosing of orally administered 30 mg of CAB, the steady‐state PK data of 30 mg of CAB administered once daily to study participants (n = 43) was reported by Spreen et al.^40^

The clinical data used for validating model predictions for oral RPV plasma concentrations included historical steady‐state PK data of 25 mg RPV once daily in healthy subjects (n = 12).^47^ Similarly, Ford et al reported steady‐state PK data for 25 mg of RPV administered orally once daily to two separate cohorts (n = 16 for cohort 1 and n = 12 for cohort 2) of healthy subjects.^48^ Crauwels et al also reported steady‐state data for 25 mg of RPV taken once daily by healthy subjects (n = 20).^49^ Lastly, steady‐state PK data for 150 mg of RPV once daily was also used.^50^ For RAL, the clinical data used was the steady‐state PK data of healthy postpartum women (n = 38) taking an oral dose of 400 mg once daily.^51^

LAI regimens tested in adults included 800 mg of IM CAB followed by 200 mg of IM CAB monthly, 800 mg of IM CAB followed by 400 mg of IM CAB monthly and 800 mg of IM CAB every 3 months, and 1200 mg of IM RPV followed by 600 mg of IM RPV monthly and 1200 mg of IM RPV followed by 900 mg of IM RPV monthly. Clinical data for repeated LAI CAB and LAI RPV administration in healthy adults were obtained from the study reported by Spreen et al.^40^

The PK parameters of each drug were simulated and compared with the corresponding adult data from clinical studies available in literature to validate the adult PBPK model. The doses, regimens and routes of drug administration were modelled to mimic the clinical studies used to validate the model. The model validation process was conducted in line with the EMA guidelines for PBPK model validation.^52^ Each model was considered validated when the summary statistics (mean or median) of the simulated PK parameters such as AUC, *C*_min_ and *C*_max_ were less than two‐fold of the reported clinical values and the absolute average fold error (AAFE) was also less than two.

### 2.7 Model modifications to develop a pregnancy PBPK model

Following successful validation of the nonpregnant adult model, the adult PBPK model was feminized by limiting the values for gender‐specific parameters (eg, organ weights) to female only.^30^ The adult female PBPK model was later modified to represent a pregnant population. Pregnancy‐induced anatomical, physiological and metabolic changes, known to influence PKs, were incorporated into the adult female model to generate a pregnancy PBPK model.^18^, ^53^ Blood‐flow rates to different organs and tissues, such as the muscle tissue during pregnancy, were computed as fractions of the cardiac output and were obtained from the literature.^53^ Key pregnancy‐related biological changes that were implemented in the model have been listed in Table 2. In addition, UGT1A1 activity was assumed to be induced by 1.33‐, 1.75‐ and 1.92‐fold at the first, second and third trimesters of pregnancy, respectively.^55^ The varying levels of plasma proteins were also modelled using previously established equations^26^, ^42^ to capture the effect on the unbound fraction of drug in plasma.

**TABLE 2:** Key pregnancy‐induced anatomical, physiological and metabolic changes implemented in the pregnancy model

Parameter	Equation	Reference
Body weight (kg)	Body weight = 61.1 + 0.2409GA + 0.0038GA^2^	^18^
Cardiac output (L/h)	Cardiac output = 301 + 5.916GA − 0.088GA^2^	^18^
Plasma proteins (g/L)	Plasma proteins = 69.7 + 0.2085GA − 0.0305GA^2^ + 0.0006GA^3^	^18^
CYP3A4 enzyme activity	CYP3A4 activity = X1.6	^54^

Abbreviations: CYP, cytochrome P450; GA, gestational age in pregnancy (weeks).

The clinical steady‐state PK data of oral 400 mg RAL in pregnancy were obtained from the study by Watts et al, who reported PK data in pregnant adult women in the second (n = 16) and third (*n* = 41) trimesters.^51^ Conversely, steady‐state PK data of oral 25 mg RPV in pregnancy were obtained from two studies: Osiyemi et al reported PK data in pregnant adult women in the second (n = 15) and third (n = 13) trimesters,^22^ and Schalkwijk et al reported PK data in pregnant adult women (n = 16) in the third trimester.^24^ By extension, the activities of UGT1A1 and CYP3A4 during pregnancy, as represented within the pregnancy PBPK model, were also validated in the process. Lastly, clinical data on the terminal PKs of LAI CAB and LAI RPV in pregnancy were also used to validate the pregnancy PBPK model.^23^

### 2.8 Foetal segment of pregnancy PBPK model

Drug transfer across the placenta was modelled as bidirectional passive diffusion, which has been previously described.^26^ The drug diffusion rate constant for RAL across the placenta was fitted into the model using the extensive clinical data that was available for oral RAL in pregnancy.^51^ These clinical data included the median cord blood concentrations, maternal blood concentration and median cord‐to‐maternal blood concentration ratios (C:M ratios) for RAL, unlike the clinical data available for RPV where only the median C:M ratios were reported.

Subsequently, the drug diffusion rate constant for RPV was derived using the fitted drug diffusion rate constant for RAL and the apparent permeability coefficients of RAL and RPV, as shown in Equation (6) and earlier described in literature.^26^, ^56^

(6) $$K_{\mathrm{RPV}}=\frac{P_{\mathrm{app},\mathrm{RPV}}\times K_{\mathrm{RAL}}}{P_{\mathrm{app},\mathrm{RAL}}}$$

where *K*_RAL_ and *K*_RPV_ are the drug diffusion rate constants across the placenta for RAL and RPV, respectively, and *P*_app,RAL_ and *P*_app,RPV_ are the apparent permeability coefficients of RAL and RPV, respectively. The same approach was used to estimate the drug diffusion rate constant for CAB. Available clinical data on foetal exposure to oral RPV were used to validate the estimated *K*_RPV_.^24^ CYP3A4 and UGT1A1 activities were also incorporated in the placenta and foetal liver, as earlier described by Liu et al.^45^

### 2.9 Predictions of the PKs of LAI CAB and LAI RPV in pregnancy

The two approved dosage regimens for LAI CAB comprise 600 mg of CAB administered intramuscularly followed by 400 mg of IM CAB every month (LAI CAB monthly dosing of CAB) or 600 mg of IM CAB every 2 months (LAI CAB bimonthly dosing). As for CAB, approved dosage regimens for LAI RPV include 900 mg of IM RPV for 1 month followed by 600 mg of IM RPV every month (LAI RPV monthly dosing of RPV) or 900 mg of IM RPV every 2 months (LAI RPV bimonthly dosing). In all cases, the administration of the LAI doses is preceded by their respective oral lead‐in doses for 1 month.

Simulations were performed to predict the disposition of both the monthly and bimonthly LAI CAB and LAI RPV for a total period of 12 weeks (ie, 3 months) without the oral lead‐in components. Results from a recent study suggest comparable plasma trough concentrations (*C*_trough_) after the first LAI dose with or without an oral lead‐in, although the study reported data for only CAB.^57^ The simulations in the pregnancy PBPK model were initiated in the first (weeks 1‐13), second (weeks 14‐26) and third (weeks 28‐40) trimesters of pregnancy. In addition, the female adult PBPK model was used to run similar simulations for a nonpregnant adult female. Simulations were performed to explore any differences in the disposition of both drugs if they were initiated in the first, second or third trimesters of pregnancy as compared to nonpregnant women. Predicted *C*_trough_ values were also compared against clinical target concentrations for RPV and CAB. In addition, the unbound concentrations of CAB and RPV were also predicted for the monthly dosing regimens.

An alternative dosing regimen was also explored in pregnancy for LAI CAB and LAI RPV. The alternative LAI dosing regimen was a modification of the bimonthly dosing regimens. The first dosing interval of 4 weeks between the LAI doses was maintained, after which the LAI dosing was continued every 6 weeks (in lieu of every 8 weeks of the bimonthly dosing regimen) i.e., 6‐weekly dosing 4 weeks after the first LAI dose for the alternative dosing regimen. This was performed with 600 mg of IM CAB and 900 mg of IM RPV.

C:M ratios, as a surrogate to foetal exposure, were also estimated for the monthly regimens of LAI CAB and LAI RPV during the second and third trimesters of pregnancy.

### 2.10 Sensitivity analyses

Sensitivity analyses were conducted to determine the impact of changes in the drug‐release rates (*K*_IM_) on the plasma concentrations of CAB and RPV in the pregnancy PBPK model. Similarly, the sensitivity of the C:M ratios to changes in the drug diffusion rate constant of the placenta was also evaluated.

## 3 RESULTS

### 3.1 Adult PBPK model verification

The comparison between the simulated PKs of orally administered CAB, RPV and RAL in adults against their respective observed clinical data is shown in Table 3. The AAFE values of the simulated versus observed PK parameters were all less than two‐fold, which was the accepted threshold for this study. Likewise, the simulated PKs of LAI CAB and LAI RPV in adults were compared against their corresponding clinical data, as shown in Table 4, with the AAFE yielding values below two‐fold. Thus, the adult PBPK models were considered validated and suitable for investigating the PKs of oral CAB, RPV and RAL in novel clinical scenarios. The same also applied for the suitability of the adult PBPK model to evaluate the PKs of LAI CAB and LAI RPV in adults. Supporting Information Figure S1 shows the comparison of the predicted PK curves of LAI CAB in adults with the corresponding clinical PK curve during the last dosing interval.

**TABLE 3:** Validation of adult PBPK model for oral PKs of CAB, RPV and RAL (simulated *vs* observed).

PK parameter	Observed	Simulated	AAFE
30 mg CAB (single dose)^a^, ^46^			
*C*_max_ (μg/mL)	3.61	3.07	1.18
AUC_0‐inf_ (μg·h/mL)	146	98.2	1.49
30 mg CAB (repeated doses)^a^, ^40^			
*C*_trough_ (μg/mL)	4.9	4.6	1.06
*C*_max_ (μg/mL)	8.3	7.8	1.06
AUC_0‐24_ (μg·h/mL)	147	154	1.05
25 mg RPV (repeated doses)^b^, ^47^			
*C*_min_ (ng/mL)	89.85	132.1	1.47
*C*_max_ (ng/mL)	203.8	283.2	1.39
AUC_0‐24_ (ng·h/mL)	2589	5052	1.95
25 mg RPV (repeated doses)^b^, ^49^			
*C*_trough_ (ng/mL)	67.3	123	1.82
*C*_max_ (ng/mL)	180.9	255.4	1.41
AUC_0‐24_ (ng·h/mL)	2528	4604	1.82
25 mg RPV (repeated doses)^a^, ^48^			
*C*_trough_ (ng/mL)	74.5	116	1.55
*C*_max_ (ng/mL)	148	240	1.62
AUC_0‐24_ (ng·h/mL)	2227	4340	1.95
25 mg RPV (repeated doses)^a^, ^48^			
*C*_trough_ (ng/mL)	87.4	132	1.51
*C*_max_ (ng/mL)	171	270	1.58
AUC_0‐24_ (ng·h/mL)	2473	4906	1.98
150 mg RPV (repeated doses)^b^, ^50^			
*C*_trough_ (ng/mL)	478	777	1.63
*C*_max_ (ng/mL)	1123	1694	1.51
AUC_0‐24_ (ng·h/mL)	16 051	30 055	1.87
400 mg RAL (repeated doses)^a^, ^51^			
*C*_trough_ (ng/mL)	79.7	156	1.96
*C*_max_ (ng/mL)	3035	1706	1.78
AUC_0‐12_ (ng·h/mL)	11 600	10 244	1.13

Abbreviations: AAFE, absolute average fold error; AUC_0‐12_, area under the plasma concentration time curve within 12 h; AUC_0‐24_, area under the plasma concentration time curve within 24 h; AUC_0‐inf_, area under the plasma concentration time curve till infinity; CAB, cabotegravir; *C*_max_, maximum plasma concentration; *C*_min_, minimum plasma concentration; *C*_trough_, plasma concentration at the end of the dosing interval; PBPK, physiologically‐based pharmacokinetic; PKs, pharmacokinetics; RAL, raltegravir; RPV, rilpivirine.

^a^ Data presented are geometric mean values.

^b^ Data presented are arithmetic mean values.

**TABLE 4:** Validation of adult PBPK model for PKs of LAI CAB and LAI RPV (simulated *vs* observed).

Regimen	*C*_τ_ (μg/mL)			*C*_max_ (μg/mL)			AUC_0‐τ_ (μg·h/mL)		
	Observed^a^	Simulated	AAFE	Observed^a^	Simulated	AAFE	Observed^a^	Simulated	AAFE
CAB									
800 mg IM + 200 mg IM monthly	1.61	1.68	1.04	2.2	2.0	1.09	1242	1255	1.01
800 mg IM + 400 mg IM monthly	3.27	2.71	1.21	4.4	3.3	1.35	2473	2020	1.22
800 mg IM quarterly	1.1	1.4	1.26	3.3	2.6	1.27	4467	3947	1.27

Regimen	*C*_τ_ (ng/mL)			*C*_max_ (ng/mL)			AUC_0‐τ_ (ng·h/mL)		
	Observed^a^	Simulated	AAFE	Observed^a^	Simulated	AAFE	Observed^a^	Simulated	AAFE
RPV									
1200 mg IM + 600 mg IM monthly	78.9	126.9	1.61	126	154	1.22	63 656	94 350	1.48
1200 mg IM + 900 mg IM monthly	79.1	150	1.90	168	182	1.08	74 420	111 445	1.50

*Note*: Data presented as geometric mean values.

Abbreviations: AAFE, absolute average fold error; AUC_0‐τ_, area under the plasma concentration time curve within the dosing interval; CAB, cabotegravir; *C*_max_, maximum plasma concentration; *C*_τ_, plasma concentration at the end of the dosing interval; IM, intramuscular; LAI, long‐acting injectable; PBPK, physiologically‐based pharmacokinetic; PK, pharmacokinetics; RPV, rilpivirine.

^a^ Clinical data observed by Spreen et al.^40^

### 3.2 Pregnancy PBPK model verification

A comparison of the simulated PKs of oral RAL and oral RPV in different trimesters of pregnancy against clinical PK data is summarized in Table 5. The pregnancy PBPK model adequately predicted the PK parameters for oral RAL and oral RPV in pregnancy with AAFE values less than 2. Also, the simulated versus clinical terminal half‐lives of LAI CAB and LAI RPV in pregnancy are presented in Table 5. Similarly, the pregnancy PBPK model correctly predicted the terminal kinetics of both drugs during washout in pregnancy.

**TABLE 5:** Validation of pregnancy PBPK model for RAL, CAB and RPV in pregnant women (simulated *vs* observed)

PK parameter	Observed	Simulated	AAFE
Oral RAL			
Second trimester^a^ ^,^ ^e^			
*C*_12_ (ng/mL)	62.1	53.7	1.16
*C*_max_ (ng/mL)	2250	1224	1.84
AUC_0‐12_ (ng·h/mL)	6600	6250	1.06
Third trimester^a^ ^,^ ^e^			
*C*_12_ (ng/mL)	64	37	1.73
*C*_max_ (ng/mL)	1770	1172	1.51
AUC_0‐12_ (ng·h/mL)	5400	5665	1.05
Delivery^a^ ^,^ ^e^			
C:M ratio	1.5	1.26	1.19
Cord blood (ng/mL)	154	159	1.00
Maternal blood (ng/mL)	140	167	1.19
Oral RPV			
Second trimester^b^ ^,^ ^g^			
*C*_min_ (ng/mL)	54.3	32.6	1.67
*C*_max_ (ng/mL)	121	171	1.42
AUC_0‐24_ (ng·h/mL)	1792	2326	1.30
Third trimester^b^ ^,^ ^g^			
*C*_min_ (ng/mL)	52.9	33.4	1.58
*C*_max_ (ng/mL)	123	173	1.41
AUC_0‐24_ (ng·h/mL)	1762	2354	1.34
Third trimester^c^ ^,^ ^f^			
*C*_min_ (ng/mL)	50	32	1.56
*C*_max_ (ng/mL)	110	174	1.58
AUC_0‐24_ (ng.h/mL)	1710	2351	1.37
Delivery^c^ ^,^ ^e^			
C:M ratio	0.5	0.82	1.63
LAI RPV^d^ ^,^ ^g^			
Terminal t_1/2_ (weeks)	10.7	16.6	1.55
LAI CAB^d^ ^,^ ^g^			
Terminal *t*_1/2_ (weeks)	8.99	10.0	1.12

Abbreviations: AAFE, absolute average fold error; AUC_0‐12_, area under the plasma concentration time curve within 12 h; AUC_0‐24_, area under the plasma concentration time curve within 24 h; *C*_12_, plasma concentration 12 h after dose administration; CAB, cabotegravir; *C*_max_, maximum plasma concentration; *C*_min_, minimum plasma concentration; C:M ratio, cord‐to‐maternal blood concentration ratio at delivery; LAI, long‐acting injectable; PBPK, physiologically‐based pharmacokinetic; PKs, pharmacokinetics; RAL, raltegravir; RPV, rilpivirine; Terminal *t*_1/2_, terminal half‐life.

^a^ Watts et al.^51^

^b^ Osiyemi et al.^22^

^c^ Schalkwijk et al.^24^

^d^ Patel et al.^23^

^e^ Median values.

^f^ Geometric mean values.

^g^ Arithmetic mean values.

### 3.3 Predictions of LAI CAB and LAI RPV in pregnancy

**FIGURE 2:**
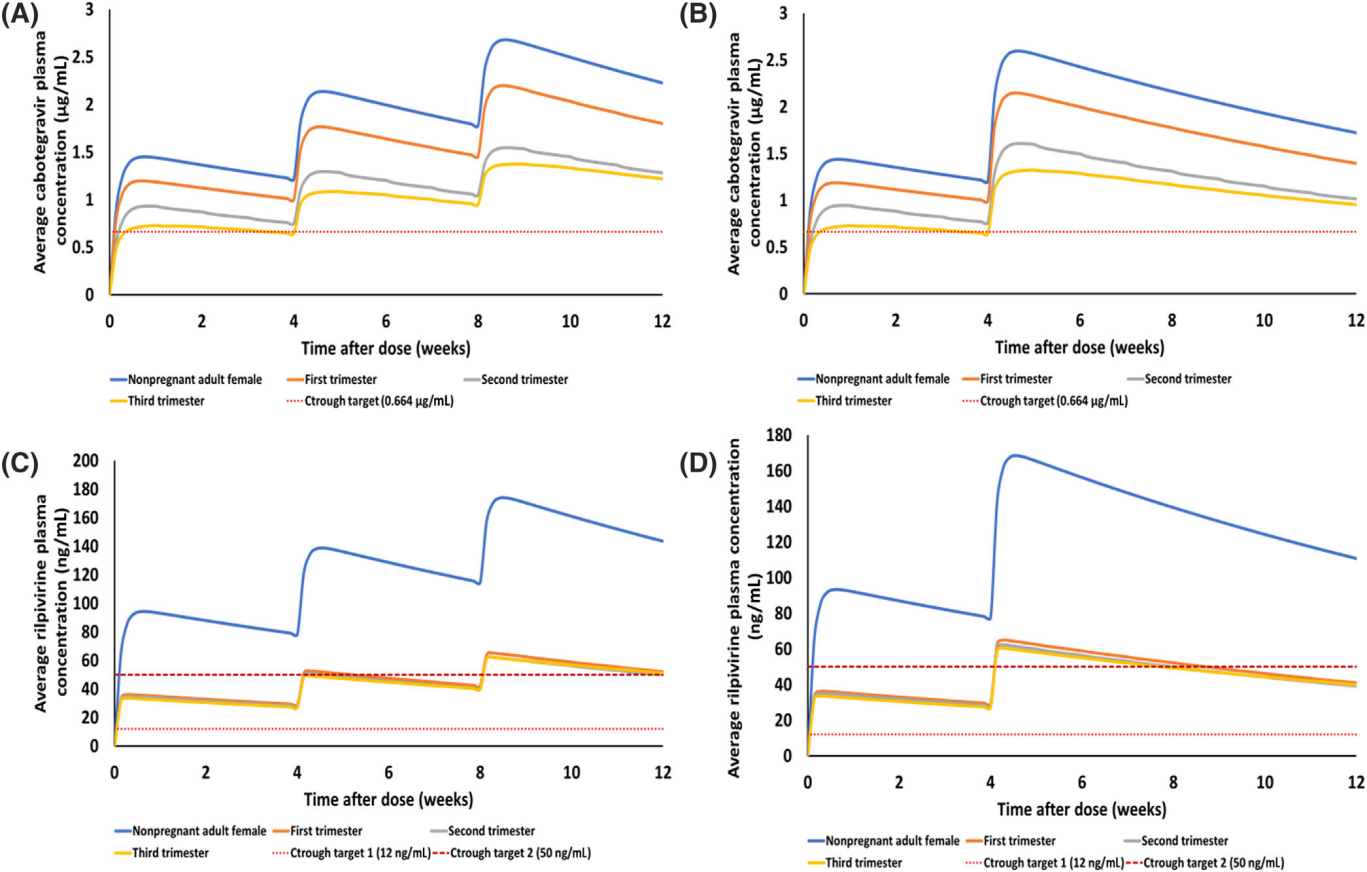
Predicted average plasma concentration‐time profile of approved dosing regimens of long‐acting injectable (LAI) cabotegravir LAI rilpivirine without oral lead‐in in pregnant and nonpregnant adults. (A) Monthly LAI cabotegravir (600 mg intramuscular [IM] cabotegravir for 4 weeks followed by 400 mg IM cabotegravir every 4 weeks), (B) bimonthly LAI cabotegravir (600 mg IM cabotegravir for 4 weeks followed by 600 mg IM cabotegravir every 8 weeks), (C) monthly LAI rilpivirine (900 mg IM rilpivirine for 4 weeks followed by 600 mg IM rilpivirine every 4 weeks) and (D) bimonthly LAI rilpivirine (900 mg IM rilpivirine for 4 weeks followed by 900 mg IM rilpivirine every 8 weeks).

The predicted PK parameters for the monthly and bimonthly dosing regimens of LAI CAB and LAI RPV in pregnant populations are shown in Figure 2. For the monthly dosing regimens of LAI CAB, *C*_trough_ of CAB at the end of 12 weeks was predicted to be higher than 4PAIC_90_ (0.664 μg/mL) throughout pregnancy (Table 6). Compared to predictions for the nonpregnant women, the predicted geometric mean ratio of the CAB *C*_trough_ was 0.81, 0.57 and 0.47 in the first, second and third trimesters of pregnancy, respectively.

**TABLE 6:** Simulated PK parameters for monthly and bimonthly dosing of LAI CAB and LAI RPV (without oral lead‐in component) at week 12 in pregnant and nonpregnant women.

		CAB 600 mg then 400 mg monthly^a^ (n = 400)			CAB 600 mg bimonthly^b^ (n = 400)				RPV 900 mg then 600 mg monthly^a^ (n = 400)			RPV 900 mg bimonthly^b^ (*n* = 400)		
		*C*_trough_ (μg/mL)	*C*_max_ (μg/mL)	AUC_0‐672_ (μg·h/mL)	*C*_trough_ (μg/mL)	*C*_max_ (μg/mL)	AUC_0‐1344_ (μg·h/mL)		*C*_trough_ (ng/mL)	*C*_max_ (ng/mL)	AUC_0‐672_ (ng·h/mL)	*C*_trough_ (ng/mL)	C_max_ (ng/mL)	AUC_0‐1344_ (ng·h/mL)
Non‐pregnant women	Geometric mean	2.20	2.65	1632	1.70	2.57	2857	Geometric mean	142	172	105 703	110	167	184 491
	%CV	53.7	44.4	0.07	68.9	45.4	0.04	%CV	0.82	0.67	0.001	1.07	0.70	0.001
	% C_trough_ < 0.664 μg/mL	0	‐	‐	0	‐	‐	% *C*_trough_ < 50 ng/mL	0	‐	‐	0	‐	‐
First trimester	Geometric mean	1.78	2.18	1332	1.38	2.12	2341	Geometric mean	52	64	39 075	41	64	69 782
	%CV	65.2	53.1	0.09	85.8	55.4	0.05	%CV	2.16	1.73	0.003	2.74	1.72	0.002
	% C_trough_ < 0.664 μg/mL	0	‐	‐	0	‐	‐	% *C*_trough_ < 50 ng/mL	40	‐	‐	98	‐	‐
Second trimester	Geometric mean	1.27	1.53	944	1.00	1.59	1726	Geometric mean	48	62	37 130	39	62	66 795
	%CV	91.4	75.4	0.12	116	73.2	0.07	%CV	2.25	1.81	0.003	2.85	1.8	0.002
	% C_trough_ < 0.664 μg/mL	0	‐	‐	0	‐	‐	% *C*_trough_ < 50 ng/mL	54	‐	‐	99	‐	‐
Third trimester	Geometric mean	1.21	1.36	871	0.94	1.31	1532	Geometric mean	51	62	37 648	40	60	66 038
	%CV	95.9	83.5	0.13	123	87.7	0.08	%CV	2.22	1.83	0.003	2.83	1.87	0.002
	% C_trough_ < 0.664 μg/mL	0	‐	‐	0.5	‐	‐	% *C*_trough_ < 50 ng/mL	45	‐	‐	97	‐	‐

Abbreviations: AUC_0‐672_, area under the plasma concentration time curve within the last dosing interval period of 1 month (672 h); AUC_0‐1344_, area under the plasma concentration time curve within the last dosing interval period of 2 months (1344 h); CAB, cabotegravir; *C*_max_, maximum plasma concentration within the dosing interval; *C*_trough_, plasma concentration at the end of the dosing interval; CV, coefficient of variation; LAI, long‐acting injectable; PK, pharmacokinetic; RPV, rilpivirine.

^a^ PK parameters between weeks 9 and 12 of drug administration.

^b^ PK parameters between weeks 5 and 12 of drug administration.

Conversely, for the bimonthly dosing of LAI CAB, CAB *C*_trough_ was only predicted to be lower than 4PAIC_90_ in 0.5% of the pregnant population during the third trimester of pregnancy. Compared to predictions for nonpregnant women, the predicted geometric mean ratio of the CAB *C*_trough_ was 0.81, 0.57 and 0.55 in the first, second and third trimesters of pregnancy, respectively.

Although the predicted RPV *C*_trough_ at the end of 12 weeks was higher than 12 ng/mL (EC_90_) throughout pregnancy with the monthly dosing regimen, the predicted RPV *C*_trough_ was lower than 50 ng/mL in 40%, 54% and 45% of the pregnant population in the first, second and third trimesters of pregnancy, respectively (Table 6). Compared to predictions for nonpregnant women, the predicted geometric mean ratio of the RPV *C*_trough_ was 0.37, 0.34 and 0.36 in the first, second and third trimesters of pregnancy, respectively.

Similar to the LAI RPV monthly dosing, the predicted RPV *C*_trough_ for the bimonthly dosing of LAI RPV was lower than 50 ng/mL in 98%, 99% and 97% of the pregnant populations in the first, second and third trimesters of pregnancy, respectively (Table 6). However, the predicted *C*_trough_ was also above the protein‐adjusted EC_90_ for RPV (12 ng/mL) throughout pregnancy for the bimonthly dosing regimen of RPV.

With the alternative dosing regimens explored for LAI RPV and LAI CAB, the predicted CAB *C*_trough_ was greater than 0.664 μg/mL at week 10 throughout pregnancy (Supporting Information Table S1). However, the predicted RPV *C*_trough_ was still lower than 50 ng/mL in over 80% of the pregnant population throughout pregnancy.

**FIGURE 3:**
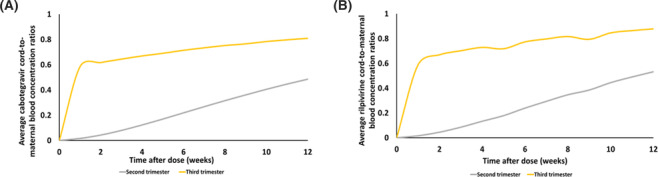
Predicted average cord‐to‐maternal blood concentration ratios during monthly dosing in pregnancy: (A) cabotegravir and (B) rilpivirine.

Foetal exposure to LAI CAB and LAI RPV was predicted to increase with pregnancy for both drugs. Predicted C:M ratios for LAI CAB and LAI RPV with the monthly dosing regimens are shown in Figure 3. No difference was observed between the predicted C:M ratios with monthly dosing and bimonthly dosing (data not shown). In addition, predicted C:M ratios were less than 1.00 throughout pregnancy for LAI RPV, but as high as 1.68 for LAI CAB in the third trimester of pregnancy.

Predicted unbound plasma concentrations of CAB and RPV in pregnancy are shown in Supporting Information Figure S2 for the monthly dosing of LAI CAB and LAI RPV. Pregnancy was predicted to affect the unbound plasma concentrations for both CAB and RPV. However, the degree of variation across pregnancy was lesser for the unbound plasma concentrations of RPV compared with CAB.

### 3.4 Sensitivity analyses

Sensitivity analyses show that the model predictions of the plasma concentrations of CAB and RPV are significantly affected by the drug release rates (Supporting Information Figure S3). Similarly, predictions of the cord‐to‐maternal blood concentration ratios for LAI CAB and LAI RPV were affected by changes to the drug diffusion rate constants for both drugs (Supporting Information Figure S4). However, changes to the drug diffusion rate constants did not significantly affect the predicted maternal plasma concentrations for both drugs (data not included).

## 4 DISCUSSION

We successfully developed a pregnancy PBPK model to predict the disposition of the approved monthly and bimonthly dosing regimens of LAI CAB and LAI RPV in pregnancy without the oral lead‐in components. An earlier study had shown that *C*_trough_ values after the first LAI CAB dose with or without an oral lead‐in were comparable, although the target *C*_trough_ could be achieved faster with the LAI CAB dose if given with the oral lead‐in component.^57^ The pregnancy PBPK model was developed from a validated adult PBPK model by incorporating pregnancy‐induced biological changes that are known to influence PKs such as changes in body weight, relevant enzyme activities and cardiac output defined by gestational age.^18^ The adult PBPK model was validated with the PK data of LAI CAB and LAI RPV in adults.^40^ Similarly, available clinical PK data of oral RPV in pregnancy were used to validate the pregnancy PBPK model for RPV PK and, by extension, the CYP3A4 activity in pregnant women. The absence of clinical PK data for oral CAB in pregnancy led to the adoption of a probe substrate (RAL) to validate UGT1A1 activity in pregnancy. RAL was considered a suitable probe substrate in this instance as it is solely metabolized by UGT1A1 and clinical PK data for oral RAL in pregnancy are available.^51^ The use of a probe substrate to validate enzyme activity for a different drug with inadequate data has been previously reported in another study.^58^ In addition, model predictions for LAI CAB and LAI RPV in pregnancy were compared against available clinical data on the terminal PKs for both drugs during washout in pregnancy.^23^

Model predictions suggest a monthly dosing regimen of LAI CAB is able to maintain efficacy in pregnancy, particularly between the first and second LAI dose (Table 6). Similarly, bimonthly dosing of LAI CAB was predicted to maintain efficacy in the majority of the pregnant population by week 12. However, there might be need for caution with the bimonthly regimen of CAB during the third trimester of pregnancy. Careful clinical evaluation, including viral load monitoring and potentially therapeutic drug monitoring, might be helpful in this regard. Alternatively, reduction of the bimonthly dosing interval from 8 to 6 weeks might help to ensure drug concentrations are maintained above the *C*_trough_ target for LAI CAB in pregnancy for all patients. In contrast, the predicted RPV *C*_trough_ at week 12 with the monthly dosing regimen of LAI RPV was below the RPV *C*_trough_ target in at least 40% of the pregnant population in the first, second and third trimesters of pregnancy. In addition, the predicted RPV *C*_trough_ at week 12 with the bimonthly dosing regimen of LAI RPV was below the RPV *C*_trough_ target in over 90% of the pregnant population throughout pregnancy. Hence, there might be a need for caution with the introduction of monthly and bimonthly regimens of RPV throughout pregnancy. However, we are unsure if the effect of pregnancy on the PKs of LAI RPV would be clinically significant if steady state had been reached before the pregnancy began.

Predicted C:M ratios increased with gestation for both drugs during pregnancy (Figure 3). The median RPV C:M ratio was predicted to be 0.88 (range 0.78‐0.93) between weeks 38 and 40 of pregnancy with monthly LAI RPV administration. Osiyemi et al^22^ reported a median C:M ratio of 0.55 (range 0.43‐0.98) for oral RPV at delivery while Schalkwijk et al^24^ reported a median C:M ratio of 0.5 (range 0.35‐0.81) for oral RPV, also at delivery. For LAI CAB, the median CAB C:M ratio was predicted as 1.71 (range 1.55‐1.79) between weeks 38 and 40 of pregnancy. Currently, there are no reported C:M ratios for CAB from pregnant women at delivery, but an ex‐vivo cotyledon study estimated median foetal‐to‐maternal exposure to CAB as 0.1.^59^ In addition, predicted foetal exposure to both LAI CAB and LAI RPV was highly sensitive to changes in the drug diffusion rate constants implemented in the pregnancy PBPK model to estimate transplacental drug transfer (Supporting Information Figure S4). Unbound plasma concentrations of CAB and RPV were also predicted to be affected by pregnancy. However, the effect of pregnancy on the unbound plasma concentrations was predicted to be greater for CAB than RPV. This might be explained by differences in their binding to plasma proteins.^43^

In the PBPK model, a simple first‐order equation was used to characterize the absorption/release rate of the drug into the systemic circulation from the IM depot. The mathematical expression was independent of the size of the patient's muscle mass, which could explain why the predicted PKs of LAI CAB did not vary significantly between virtual patients with different body mass indices (BMIs). Unlike the predicted PKs of LAI CAB, studies in humans have reported that BMI is a significant covariate for the PKs of LAI CAB.^60^ The size of muscle mass might affect the available depot space for the drug in the muscle, which could lead to a faster release of the drug into the systematic circulation and contribute to a faster decline of the LAI CAB concentrations in patients with low BMI.^60^, ^61^ Patel et al reported higher maximal levels of LAI CAB in the plasma of a study volunteer with lower BMI compared to two others with higher BMI.^61^ However, the release rates of LAI CAB and LAI RPV used in this study were fitted into the model with available clinical data.^39^, ^40^ It is also important to consider that changes to the rate of blood flow through the muscle tissue might affect the release rate of the drug from the IM depot, whereas a fixed drug release rate was used in our pregnancy PBPK model. Cardiac output has been reported to vary with gestational age, so the rate of blood flow to the muscle tissue is also expected to vary during pregnancy.^18^, ^53^ However, there is currently inadequate data to characterize any relationship between changes in blood flow through the muscle tissue and the drug release rate from the muscle. Thus, it is remains unknown if changes in blood flow rates to the muscle during pregnancy would result in significant differences in drug release rates. Sensitivity analyses of the plasma concentrations of CAB and RPV to variations of their release rates are shown in Supporting Information Figure S1. The PKs of monthly and bimonthly LAI CAB and LAI RPV were also simulated for nonpregnant adult females for comparison with the pregnant population because LAI CAB PK has been reported to differ between males and females.^60^

One major limitation of this study is the inability to simulate the continuation of the PKs of the LAI drugs from nonpregnant women into pregnancy. Separate models were developed for the nonpregnant and pregnant population owing to the complexity of incorporating the effect of gestation on parameters affected by pregnancy. In the clinic, it might be more likely for nonpregnant women receiving LAI CAB and LAI RPV to become pregnant and choose to continue with the injectable formulations than for treatment‐naïve women to be initiated with the injectables when they have also been confirmed to be pregnant. Despite this limitation, this study has been able to demonstrate the likely impact of pregnancy on the PKs of LAI CAB and LAI RPV. For women already receiving the LAIs before becoming pregnant, model predictions suggest they are less likely to have subtherapeutic plasma concentrations of CAB with the monthly dosing regimen if they have completed at least one dosing cycle of the LAIs or achieved steady state before becoming pregnant.

Another limitation of this study is the use of an orally administered probe substrate (RAL) of UGT1A with a different mode of administration compared to the study drug in the absence of adequate clinical data in pregnancy for other long‐acting drugs that are substrates for UGT1A. Likewise, drug transporter activity was not included in the PBPK model, but this was primarily due to lack of relevant data. RPV is not a known substrate of any drug transporter. On the other hand, CAB is a substrate of multidrug resistance protein 1 (P‐glycoprotein 1) and breast cancer resistance protein (BCRP) in vitro.^62^ Although pregnancy has been reported to influence the activity of P‐glycoprotein 1 and BCRP in rodents,^63^, ^64^ data in humans are not available. Regardless, the influence of drug transporter activity on the PKs of oral CAB appear to be minimal.^62^

The administration of LAI drugs in pregnancy is not a new paradigm. LAI antipsychotic drugs have been administered in pregnancy for over two decades. Despite this long duration, PK data on the use of LAI antipsychotics in pregnancy have been very limited.^65^ Similarly, outcomes on the safety of LAI antipsychotics in pregnancy have been inconsistent. Where poor outcomes have been reported in pregnancy after the use of LAI antipsychotics, there have been insufficient data to determine if the poor outcomes are due to the illness, class of drug or the long‐acting formulation.^66^ Nonetheless, there have been strong arguments for LAI antipsychotic use during pregnancy owing to improved adherence, reduced risk of overdose and less psychiatric rehospitalisation compared to oral antipsychotics.^67^ Adherence to antipsychotics is particularly important during pregnancy to prevent relapses which might lead to poor birth outcomes.^67^

In a similar vein, adherence to antiretrovirals in pregnancy is highly necessary to reduce the risk of vertical transmission of HIV. LAI antiretrovirals might be a preferred choice throughout pregnancy to support adherence and to reduce psycho‐social challenges relating to disclosure of HIV status. In addition, the new option of LAI antiretrovirals might be particularly important in early pregnancy for women living with HIV who may prefer a non‐oral route of drug administration due to nausea and vomiting. However, there is a need to frequently monitor pregnant women on LAI antiretrovirals towards improving available data on safety and efficacy. PBPK modelling readily overcomes the many ethical and logistic challenges associated with randomized clinical trials in complex populations. It could also prove useful in exploring PKs in complex clinical scenarios and complex populations.

## 5 CONCLUSION

Since the approval of LAI RPV and LAI CAB for the general adult population, there have been limited clinical data to guide the dosing of both LAIs in pregnant women. In this study, we developed a pregnancy PBPK model to describe plasma concentrations of LAI CAB and LAI RPV in pregnancy. Based on the model predictions, both the monthly and bimonthly dosing regimen of LAI CAB could maintain antiviral efficacy throughout pregnancy without need for adjustments. However, both monthly and bimonthly dosing regimens of LAI RPV might be better not introduced in pregnancy, although they might be continued if steady state had been reached with caution and adequate monitoring. Future clinical studies in humans are needed to confirm these model predictions.

## AUTHOR CONTRIBUTIONS

S.A., C.W. and M.S. contributed to the study conception. All authors contributed to the overall design of the study, with specific input from A.O. on modelling the foetal compartment. S.A. performed the modelling analyses. The first draft of the manuscript was written by S.A. and all other authors commented on previous versions of the manuscript. All authors reviewed and contributed to the final manuscript.

## CONFLICT OF INTEREST STATEMENT

M.S. has received research grant funding from Janssen and ViiV unrelated to this work. M.S. and F.B. are currently employed by Labcorp and M.C.M. is currently employed by Pfizer. All other authors have no potential conflicts of interest to declare.

## Supporting information


**SUPPORTING INFORMATION TABLE S1.** Simulated PK parameters for the alternative dosing regimen of LAI CAB and LAI RPV (without oral lead‐in component) at week 10 in pregnant women.

**SUPPORTING INFORMATION FIGURE S1.** Predicted PK curves of LAI CAB in adults *vs* corresponding clinical PK curves during the last dosing interval. LAI CAB was administered (A) at 800 mg IM CAB followed by 200 mg IM CAB monthly, (B) at 800 mg IM CAB followed by 400 mg IM CAB monthly and (C) at 800 mg IM CAB administered every 3 months. Clinical data were reported by Spreen et al (2014).

**SUPPORTING INFORMATION FIGURE S2.** Predicted average unbound plasma concentrations with monthly dosing in pregnancy: (A) cabotegravir and (B) rilpivirine.

**SUPPORTING INFORMATION FIGURE S3.** Sensitivity analyses of plasma concentrations of (A) rilpivirine and (B) cabotegravir to variations (±25%, 50% and 75%) of their respective release rates from the long‐acting injectable formulations in the pregnancy PBPK model. The sensitivity analysis was performed for single doses of 600 mg of long‐acting rilpivirine and 600 mg of long‐acting cabotegravir.

**SUPPORTING INFORMATION FIGURE S4.** Sensitivity analyses of cord‐to‐maternal blood concentration ratios of (A) rilpivirine and (B) cabotegravir to variations (±25%, 50% and 75%) of their respective drug diffusion rate constants in the pregnancy PBPK model. The sensitivity analysis was performed for single doses of 600 mg of long‐acting rilpivirine and 600 mg of long‐acting cabotegravir.

## Data Availability

The data that support the findings of this study are available from the corresponding author upon reasonable request.
