# Supplementary material for: Physiologically‐based pharmacokinetic modelling of long‐acting injectable cabotegravir and rilpivirine in pregnancy
Source: Br J Clin Pharmacol. 2024 Feb 10;91(4):989–1002. doi: 10.1111/bcp.16006 (PMC11992663; doi:10.1111/bcp.16006)
Supplement: Supplementary file 1 — SUPPORTING INFORMATION TABLE S1. Simulated PK parameters for the alternative dosing regimen of LAI CAB and LAI RPV (without oral lead‐in component) at week 10 in pregnant women. [file BCP-91-989-s002.docx]

Table S1: Simulated PK parameters for the alternative dosing regimen of LAI CAB and LAI RPV (without oral lead-in component) at week 10 in pregnant women.

|  | ***First trimester*** | ***Second trimester*** | ***Third trimester*** |
| --- | --- | --- | --- |
| ***CAB 600mg 6-weekly after first LAI dose (n=400)*** |  |  |  |
| *% C_trough_ <0.664 μg/ml* | 0 | 0 | 0 |
| ***RPV 900mg 6-weekly after first LAI dose (n=400)*** |  |  |  |
| *% C_trough_ <50 ng/ml* | 83 | 88 | 88 |

PK parameters are between week 5-10 of drug administration; C_trough_ – plasma concentration at the end of the dosing interval.
