# Supplementary figures and images for: Physiologically‐based pharmacokinetic modelling of long‐acting injectable cabotegravir and rilpivirine in pregnancy
Source: Br J Clin Pharmacol. 2024 Feb 10;91(4):989–1002. doi: 10.1111/bcp.16006 (PMC11992663; doi:10.1111/bcp.16006)

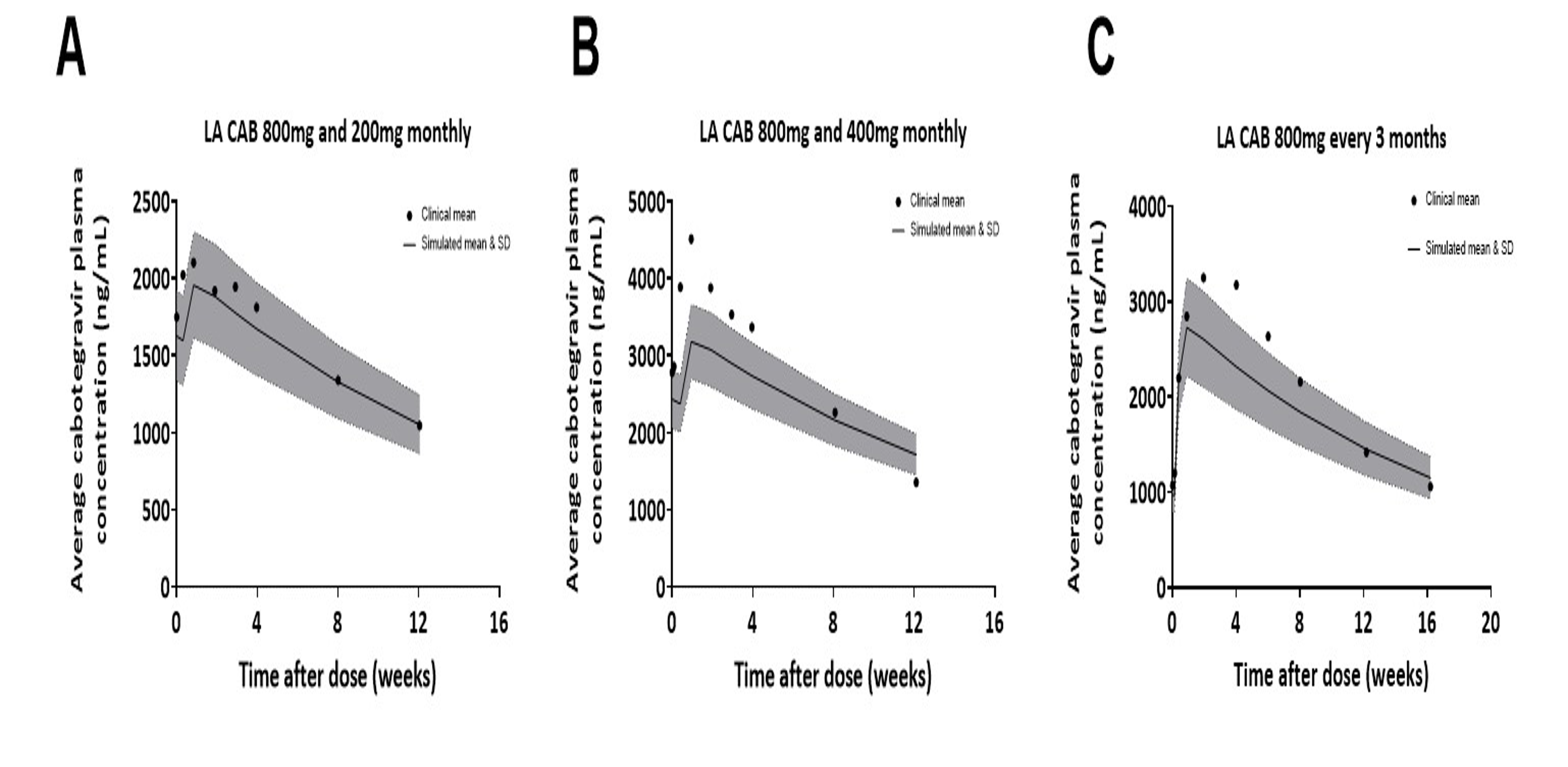

Supplement: Supplementary file 2 — SUPPORTING INFORMATION FIGURE S1. Predicted PK curves of LAI CAB in adults vs corresponding clinical PK curves during the last dosing interval. LAI CAB was administered (A) at 800 mg IM CAB followed by 200 mg IM CAB monthly, (B) at 800 mg IM CAB followed by 400 mg IM CAB monthly and (C) at 800 mg IM CAB administered every 3 months. Clinical data were reported by Spreen et al (2014). [file BCP-91-989-s001.png]

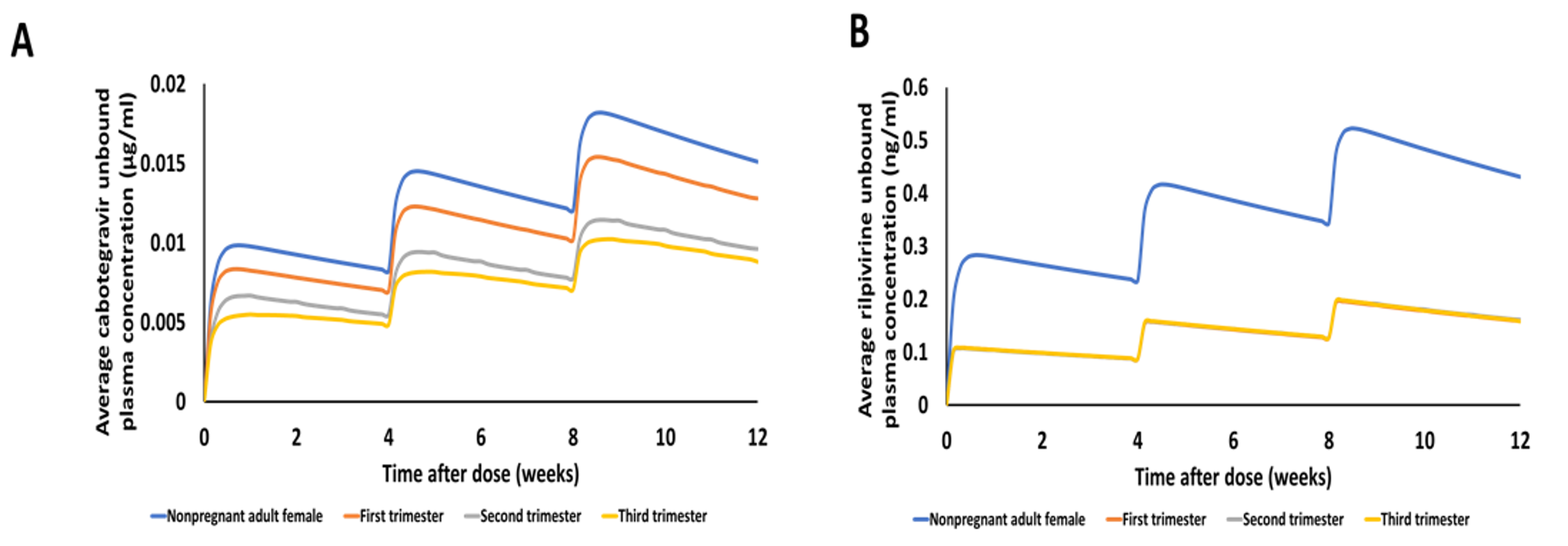

Supplement: Supplementary file 3 — SUPPORTING INFORMATION FIGURE S2. Predicted average unbound plasma concentrations with monthly dosing in pregnancy: (A) cabotegravir and (B) rilpivirine. [file BCP-91-989-s005.png]

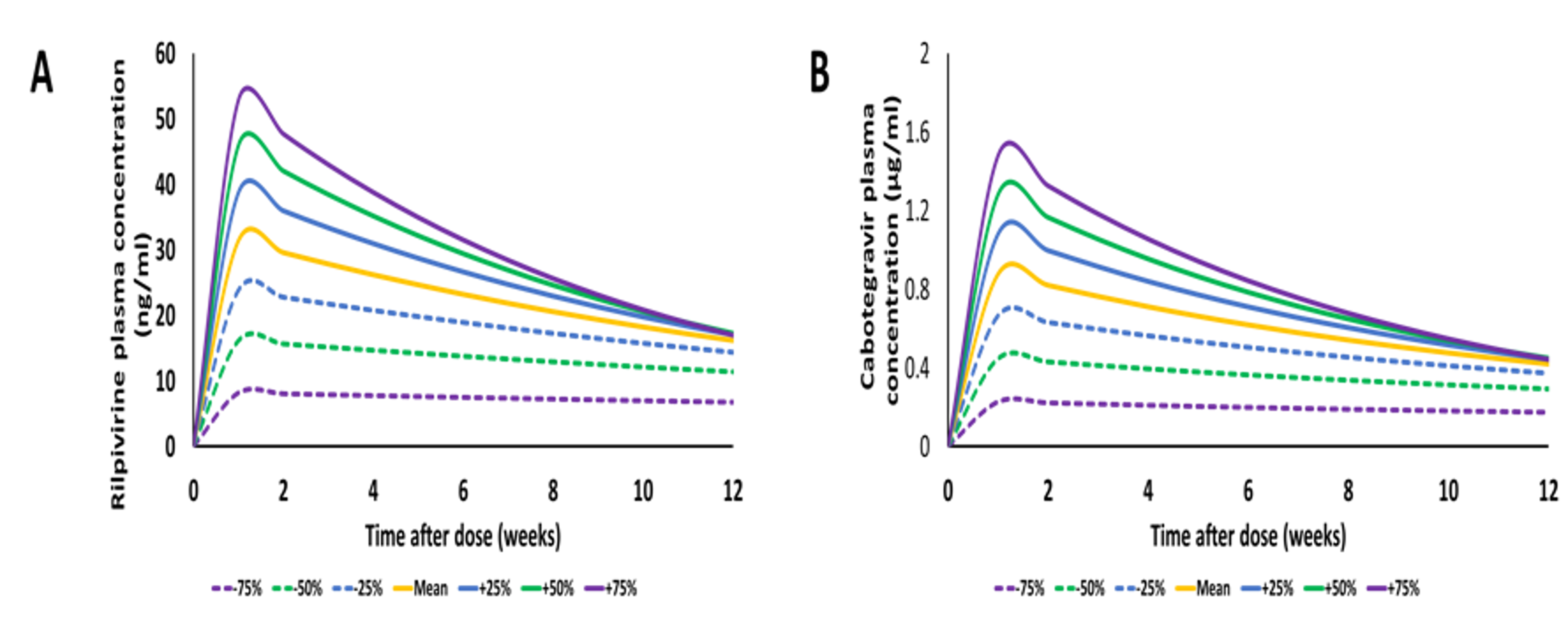

Supplement: Supplementary file 4 — SUPPORTING INFORMATION FIGURE S3. Sensitivity analyses of plasma concentrations of (A) rilpivirine and (B) cabotegravir to variations (±25%, 50% and 75%) of their respective release rates from the long‐acting injectable formulations in the pregnancy PBPK model. The sensitivity analysis was performed for single doses of 600 mg of long‐acting rilpivirine and 600 mg of long‐acting cabotegravir. [file BCP-91-989-s003.png]

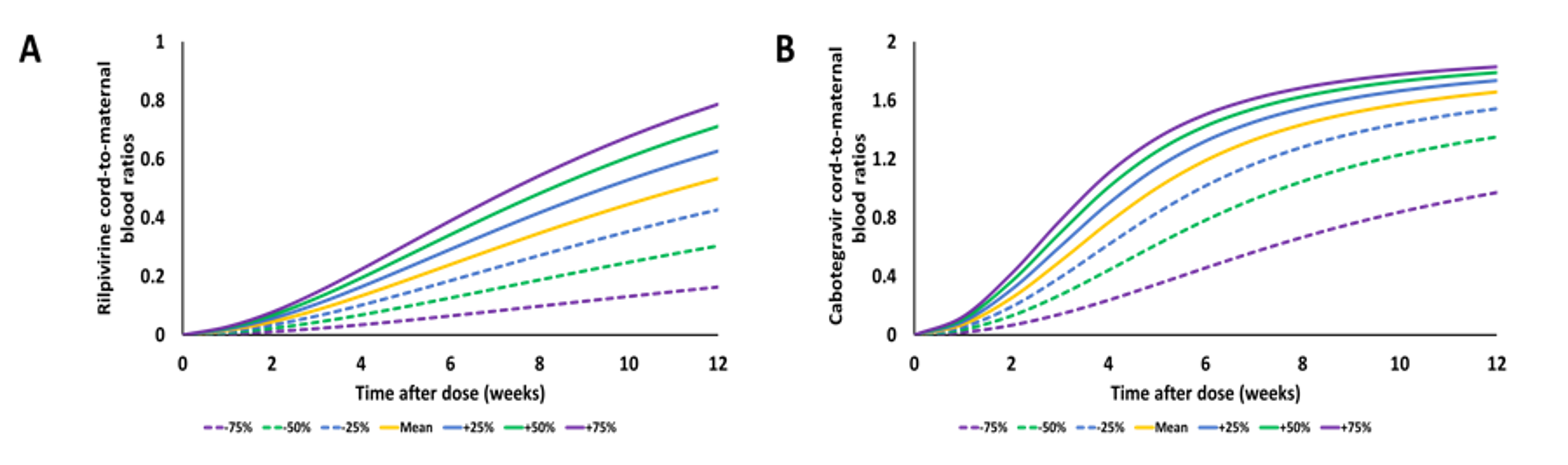

Supplement: Supplementary file 5 — SUPPORTING INFORMATION FIGURE S4. Sensitivity analyses of cord‐to‐maternal blood concentration ratios of (A) rilpivirine and (B) cabotegravir to variations (±25%, 50% and 75%) of their respective drug diffusion rate constants in the pregnancy PBPK model. The sensitivity analysis was performed for single doses of 600 mg of long‐acting rilpivirine and 600 mg of long‐acting cabotegravir. [file BCP-91-989-s004.png]
